# Sudden Cell Death Induced by Ca^2+^ Delivery via Microbubble Cavitation

**DOI:** 10.3390/biomedicines9010032

**Published:** 2021-01-04

**Authors:** Martynas Maciulevičius, Diana Navickaitė, Sonam Chopra, Baltramiejus Jakštys, Saulius Šatkauskas

**Affiliations:** Biophysical Research Group, Faculty of Natural Sciences, Vytautas Magnus University, Vileikos st. 8, LT-44404 Kaunas, Lithuania; diana.navickaite@vdu.lt (D.N.); sonam.chopra@vdu.lt (S.C.); baltramiejus.jakstys@vdu.lt (B.J.)

**Keywords:** ultrasound, sonoporation, microbubbles, calcium, cavitation

## Abstract

Intracellular calcium ion delivery via sonoporation has been validated to be a substitute for classical chemotherapy. However, the mechanism behind calcium sonoporation remains unclear to this day. To elucidate the role of calcium in the process of sonoporation, we aimed to investigate the influence of different calcium concentration on cell membrane permeabilization and cell viability after sonoporation. In this study, we present experimental evidence that extracellular calcium plays a major role in cell membrane molecular transport after applying ultrasound pulses. Ultrasound-microbubble cavitation in the presence of different calcium concentration affects fundamental cell bio-physio-chemical conditions: cell membrane integrity, metabolic activity, and colony formation. Corresponding vital characteristics were evaluated using three independent viability tests: propidium iodide assay (20 min–3 h), MTT assay (48 h), and cell clonogenic assay (6 d). The results indicate instant cell death, as the level of cell viability was determined to be similar within a 20 min–48 h–6 d period. Inertial cavitation activities have been determined to be directly involved in calcium delivery via sonoporation according to high correlation (R^2^ > 0.85, *p* < 0.01) of inertial cavitation dose with change in either cell membrane permeabilization, metabolic activity, and colony formation efficiency. In general, calcium delivery via sonoporation induces rapid cell death, occurring within 20 min after treatment, that is the result of ultrasound mediated microbubble cavitation.

## 1. Introduction

Cell sonoporation (SP) is a physical method, developed to attain spatiotemporally regulated intracellular transfer of bioactive compounds. SP process is intrinsically related to microbubble (MB) cavitation engendered by irradiation with ultrasound (US). Simultaneous US–MB interaction leads to temporal increase in cell membrane permeability for exogenous therapeutic agents, such as anticancer drugs, DNA, RNA, etc. The concurrent application of cytotoxic drug, gemcitabine, and SP has already been well-applied for antitumour therapy of pancreatic cancer in clinical trials [1]. Currently, cellular entry of calcium ions (Ca^2+^), facilitated by physical therapeutic agent delivery techniques, has been proposed as an option for conventional chemotherapy [2].

Ca^2+^ intracellular apportionment inside membrane-bound organelles plays an essential role in cellular response to diverse stress stimuli as well as coordination of fatal mechanisms. Substantial importance of Ca^2+^ has been demonstrated to facilitate the resealing rate of cell membrane injuries, caused by MB activity after irradiation with US [3,4]. Diffusible Ca^2+^ rapidly engages numerous feedback mechanisms in respect to specific extracellular triggers. Thus, combination of Ca^2+^ and SP for tumor treatment implies the enhanced momentum of desirable outcome. Compared to conventional chemotherapeutic agent delivery modalities, such as intravascular injection or oral administration, targeted US-MB enhanced Ca^2+^ delivery may substantially reduce adverse after-effects while sustaining efficient anticancer activity.

The delivery of anticancer drugs, bleomycin (BLM), doxorubicin (DOX), etc. via physical methods is designed to stimulate cancer cell death in vitro and inhibit/reduce tumour growth in vivo. Ueno et al. and Piron et al. have shown that cancer cell death after DOX delivery via SP was significantly initiated after 24 h post exposure [5,6]. Lee et al. have shown that, although there was immediate DOX intracellular delivery via SP, cell viability decrease due to DOX cytotoxicity was achieved only after 48 h with neither immediate nor 24-h effect after SP [7]. Wang et al. have achieved cell death due to SP using mitoxantrone after 24 h [8]. Recently it has been shown by our group that cell death after BLM delivery to cells using electroporation was substantially promoted after 36–48 h, as indicated by MTT test, performed at consistent time periods after treatment [9]. Similar results after BLM delivery via SP were obtained by Sonoda et al., who have shown that melanoma cell viability decreased 48 h after therapy [10]. In addition to this, they have shown that tumor weight was reduced 4 d after SP. Therefore, in most studies, performed using anticancer drugs, cell death is evaluated after 24–48 h [11,12,13]. These observations imply the duration, necessary to achieve cell death after chemotherapy, to be in the range of hours to days.

The development of effective and non-mutagenic intracellular delivery methods for genetic materials is a major challenge for gene therapy. It is difficult for non-viral gene delivery techniques to achieve fast and efficient transfection rate, mainly due to slow plasmid cytoplasmic migration to nucleus, followed by subsequent degradation and expression kinetics [14]. Indeed, in order to obtain maximum DNA expression efficiency, 5–6 h [15], 12 h [16], or even longer periods were necessary, as indicated by increasing GFP expression rate up to 48 or 72 h after SP [17,18,19]. 

Fast cellular response after Ca^2+^ delivery via SP would offer new opportunities for initiating effective and rapid cell death by intrinsic Ca^2+^ activated mechanisms, associated to supra-physiological Ca^2+^ concentration, as compared to slow cell death, induced by chemo- or gene therapy. Therefore, deeper understanding of the biological as well as physical mechanisms behind Ca^2+^ delivery via SP is crucial for the advancement of the current treatment modality.

With the aim to determine the role of Ca^2+^ in cell death after SP, we have exploited a simplified in vitro model—Ca^2+^ intracellular delivery into Chinese hamster ovary (CHO) cells was examined in the presence of MBs and US. Therefore, in this study we aimed to simultaneously reveal the impact of different Ca^2+^ concentrations on cell membrane integrity, cell metabolic activity, and cell colony formation efficiency after SP. Propidium iodide (PI) assay (after 20 min–3 h) was used in order to evaluate cell membrane permeability. Cell metabolic activity and cell colony formation efficiency were evaluated using MTT test (after 48 h) and cell clonogenic assay (6 d), respectively.

Generally, US induced MB cavitation is considered to be dual-nature and classified into stable cavitation (SC) and inertial cavitation (IC). Open-ended debate has been initiated considering the preeminent cavitation model, since it has been explicitly demonstrated that both SC and IC were efficient to enhance biological compound delivery [20]. Thus, in this research, we have estimated the pattern of inertial cavitation, according to the results of US induced MB cavitation acoustic emissions, obtained using passive cavitation detection.

## 2. Materials and Methods

### 2.1. Cell Line

Chinese hamster ovary (CHO) cells were cultured in DMEM (Sigma Aldrich, St. Louis, MO, USA) growth medium supplemented with 10% heat non-inactivated fetal bovine serum (Sigma Aldrich, St. Louis, MO, USA), 1% L-glutamine solution (Invitrogen Inc., Carlsbad, CA, USA), and 100 U/mL penicillin with 100 μg/mL streptomycin (Sigma Aldrich, St. Louis, MO, USA) solution. The cells were grown in monolayer in 10 cm Petri dishes (TPP, Switzerland), incubated at 37 °C in 5% CO_2_ atmosphere. The cells were harvested using trypsin-EDTA solution (Sigma Aldrich, St. Louis, MO, USA).

### 2.2. Experimental Setup

**Figure 1:**
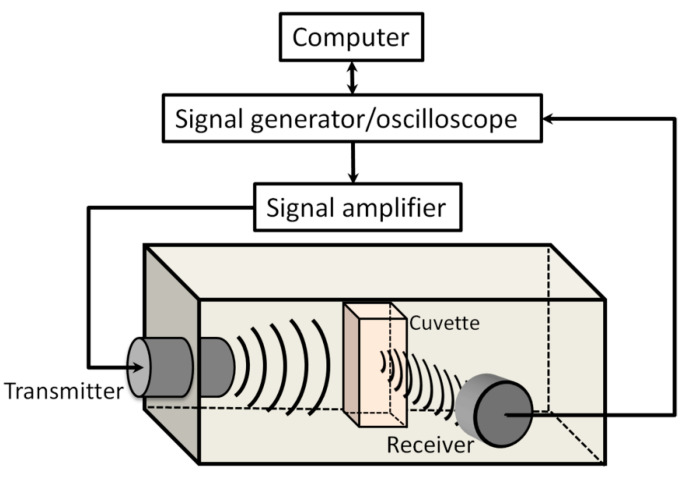
Experimental setup designed for sonoporation studies.

Sonoporation experiments were performed using experimental setup (Figure 1), consisting of experimental chamber filled with degassed water, US transducers, and signal acquisition hardware.

An arbitrary waveform generator/oscilloscope (Picoscope 5242B, Picotech, St Neots, UK) was used to generate/record US signals. The signals were amplified by signal amplifier (Kaunas University of Technology, Kaunas, Lithuania), powered by high voltage power supply (MCP Lab Electronics, Shanghai, China). The unfocused transducer (Medelkom, Vilnius, Lithuania), 18 mm diameter, operating at 1 MHz center frequency 0.9–1.2 MHz, −6 dB bandwidth, was used for MB excitation. The spatial distribution of the acoustic pressure within of US beam was evaluated to be homogenous at 1 cm distance from the excitation transducer. The US acoustic peak negative pressure in the cuvette was determined using hydrophone (HNR 1000, Onda Corp, Sunnyvale, CA, USA). In order to decrease US multiple reflections, the inner surface of the experimental chamber was covered with acoustic absorber (AptFlex F28, Precision acoustics, Dorchester, UK). The experiments were performed at room temperature (24 °C), in 1 cm width sonoporation cuvette (Plastibrand, Wertheim, Germany) filled to 1 mL volume.

Passive cavitation detection was performed using 8 mm diameter, 5 MHz center frequency 2.1–7.9 MHz, −6 dB bandwidth, transducer (Doppler Electronic Technologies, Guangzhou, China), referred as a receiver, positioned at 90° angle to the transmitter.

### 2.3. Experimental Procedure

CHO cells were sonoporated with different CaCl_2_ (Lachema, Brno, Czech Republic) concentration using US alone or US in combination with Sonovue MBs (Bracco diagnostics Inc, Manno, Switzerland). Sonovue MBs were prepared in 0.9% NaCl solution according to manufacturer’s instructions. Sonoporation experiments were performed in 0.9% NaCl supplemented with different concentration (0–20 mM) CaCl_2_ solution. MB and cell concentrations were 4 × 10^7^ MB/mL and 0.75 × 10^6^ cells/mL, respectively, yielding 53:1 MB to cell ratio. MB or cell concentration was evaluated using optical microscope (Nicon Eclipse TS100, Tokyo, Japan) and hematocytometer (Assistent, Sondheim, Germany). The experimental groups were divided depending on the type of experiment. For US alone, experimental series were (1) control group (no treatment); (2) US group (US); (3) Ca^2+^ + US group (Ca^2+^ + US). For US and MB combination, experimental series were (1) control group (no treatment); (2) cavitation group (MB + US), refers to therapeutic group at 0 mM Ca^2+^ concentration; (3) therapeutic group (Ca^2+^ + MB + US). Cells were sonoporated using 1 MHz central frequency, 1 kHz pulse repetition frequency, 10% duty cycle (100 µs on, 900 µs off), 0–800 kPa peak negative acoustic pressure US, and 6 s exposure duration. After US irradiation, the cells were incubated for 10 min at 37 °C, and then transferred to growth medium for subsequent preparation for cell viability assessment using the following assays: PI, MTT, or cell clonogenic. 

The level of bioeffects (permeabilization, metabolic activity decrease, or colony formation efficiency decrease) in (Ca^2+^ + MB + US) group can be the result of (i) cell death caused by US alone and/or US induced MB cavitation (MB + US), (ii) the cells killed by alone Ca^2+^ or MBs or their combination (Ca^2+^ + MB), (iii) Ca^2+^ sonotransfer due to reversible cell permeabilization resulting in cell death due to intracellular Ca^2+^ toxicity. Therefore, in order to reveal the change of membrane permeabilization, metabolic activity, and cell colony formation efficiency, the percentages of cell permeabilization, metabolic activity decrease, and decrease in colony formation efficiency in (MB + US) and (Ca^2+^ + MB) groups were subtracted from the corresponding percentage obtained in (Ca^2+^ + MB + US) group. Subtraction is performed in order to evaluate the effect of intracellularly delivered Ca^2+^ to cell permeability, cell metabolic activity, and cell colony formation efficiency. In the results, (Ca^2+^ + MB) group refers to (Ca^2+^ + MB + US) group at 0 kPa acoustic pressure.

### 2.4. Propidium Iodide (PI) Assay

PI assay is a cell viability assay based on the assessment of cell membrane integrity. PI intracellular uptake is due to the loss of cell membrane barrier function. After cell incubation, the medium from cell samples was removed by centrifugation. Subsequently, cells were resuspended in 1 × PBS (Lonza Inc., Rockland, ME, USA) and supplemented with PI (40 µM final concentration). The number of PI positive cells was evaluated at different time after SP: 20 min, 1 h, 2 h, and 3 h using flow cytometer (BD Accuri C6, Accuri Cytometers Inc., Ann Arbor, MI, USA).

### 2.5. MTT Assay

MTT assay is a colorimetric cell viability assay based on the assessment of cell metabolic/enzymatic activity. After incubation, 8 × 10^3^ cells were grown in the growth medium (100 µl) in the wells of 96-well microplate (Plastibrand, Wertheim, Germany) for 48 h. At the time of measurement, the growth medium was removed, and the cells in the wells were supplemented with fresh growth medium and MTT salt solution (0.5 mg/mL final concentration) and incubated for 2 h. Afterwards the medium was removed from the wells and the wells were washed with 1×PBS. Formazan formed in the cells was dissolved in isopropanol (Chempur, Karlsruhe, Germany). All the resulting content of each well was transferred into the corresponding well of another transparent 96-well microplate for absorbance measurements using microplate reader (Spectrostar Nano, BMG Labtech, Ortenberg, Germany). Optical density of the suspension was evaluated at 550 nm wavelength; the values were corrected by subtracting the background, and then normalized to the control (no treatment).

### 2.6. Cell Clonogenic Assay 

Cell clonogenic assay is a cell viability assay based on live cell ability to proliferate—form colonies. After cell incubation, 330 cells were loaded into 4.1 cm^2^ tissue culture dishes (TPP, Switzerland) containing 2 mL of growth medium. The cells were allowed to grow for 6 days, and then fixed in 1 mL of 96% ethanol for 10 min and stained using crystal violet solution (Sigma Aldrich, St. Louis, MO, USA) that contained 2.3% crystal violet, 0.1% ammonium oxalate, and 20% ethanol. The number of cell colonies was assessed using light microscope (MBS 9, LOMO, St. Petersburg, Russia) and then normalized to the control (no treatment).

### 2.7. Cavitation Signal Analysis

**Figure 2:**
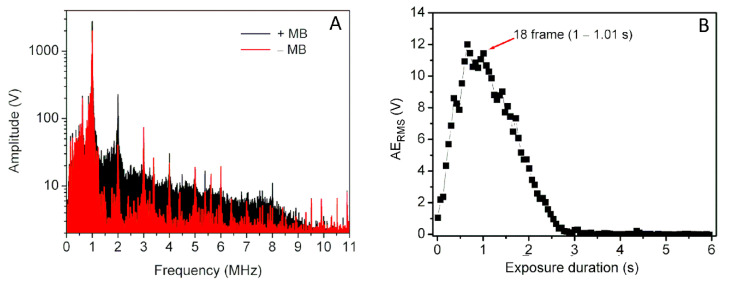
FFT of the acoustic emission pulses, recorded in the 18th frame (1–1.01 s) (**A**), corresponding estimate “Acoustic emission_RMS_ (AE_RMS_)”, quantified in 1.5–1.75 MHz frequency range (**B**), after sample exposure to 400 kPa acoustic pressure US.

US induced MB acoustic emission signals were recorded using passively coupled US transducer in order to provide information about MB cavitation activity. Passive cavitation detection system was used for MB cavitation signal detection and recording. Acoustic emission signals were acquired in two cases: (i) with MBs present (+MB group) and (ii) absent (−MB group/background group). Cavitation signals were recorded at 31.25 MS/s sampling rate, 8 bits resolution. The signals from 6 s overall exposure duration were recorded in 102 frames, corresponding to the frame rate of 17 frames/s. US signals in every frame were transformed to a frequency spectrum using fast Fourier transform (FFT). Figure 2A represents the FFT of acoustic emissions, recorded in the 18th frame at 400 kPa US excitation in two separate +MB and −MB groups. 

In order to quantitatively evaluate the amount of broadband noise, the root mean square (*RMS*) values were calculated in a particular frequency range (Equation (1)) of FFT spectrum of US acoustic emissions:

(1) $$RMS=\;\sqrt{\frac{1}{n}\left(x_{1}^{2}+x_{2}^{2}+\cdots +x_{n}^{2}\right)\;}$$

where *n* is the number of values in frequency spectrum, obtained after FFT; *x*—the amplitude value corresponding to particular frequency value (*n*).

With the aim to analyze acoustic emission signals only from MBs, background RMS values were subtracted from +MB group. Thus, the estimate “Acoustic emission_RMS_ (*AE_RMS_)*” was obtained:

(2) $$AE_{RMS}=RMS_{+MB}-\;RMS_{-MB\;}$$

where *RMS_+MB_* is *RMS*, obtained from +*MB* group and *RMS_−MB_*—from background group.

*AE_RMS_*, evaluated in the 1.5–1.75 MHz frequency range, contributed to the highest difference between +MB and background groups; therefore, it was shown in Figure 2B.

Inertial cavitation dose (*ICD*) is the integral of differential RMS in exposure duration scale [21,22]:

(3) $$ICD=\;\overset{t_{F}}{\underset{0}{\int }}AE_{RMS}\;\left(t\right)\;dt$$

where *ICD* is inertial cavitation dose, *t*-time, 0 indicates 0 s (the beginning of the exposure), and *t_F_* indicates the exposure duration when the integration is finished. 

### 2.8. Statistical Analysis

The data are presented as the mean ± standard error of the mean of at least six experimental replicates. Statistical significance between the two groups was evaluated using Student’s *t*-test, and multiple comparison was performed using one-way ANOVA. Correlation analysis was performed to determine the dependence between ICD and the sonoporation results; the strength of correlation was defined according to the correlation determination coefficient (R^2^). Data analysis was performed using Matlab (Mathworks, Natick, MA, USA) and Origin (OriginLab Co, Northampton, MA, USA) software.

## 3. Results

### 3.1. Ca^2+^ Delivery Using US Alone 

**Figure 3:**
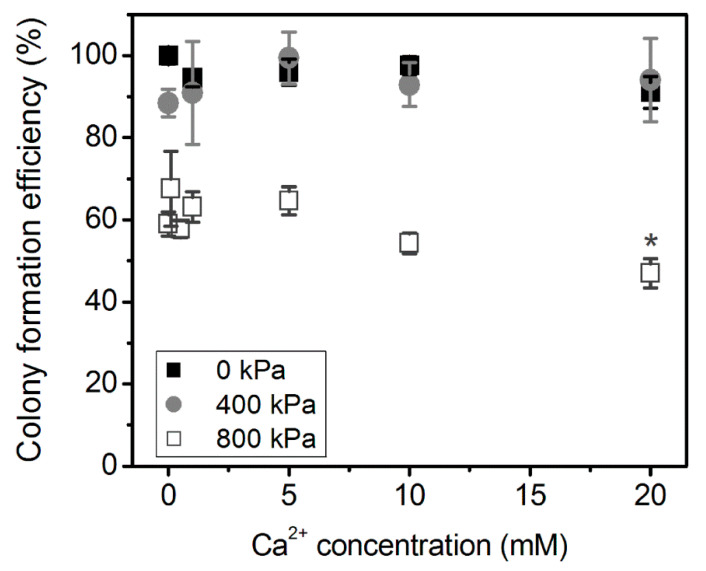
The effect of different Ca^2+^ concentration on cell colony formation efficiency: without US exposure (0 kPa) and with US exposure (400 and 800 kPa acoustic pressure). * indicates statistical significance (*p* < 0.05) between 0 and 20 mM Ca^2+^ concentration groups at 800 kPa acoustic pressure.

At the initial stage of the research, Ca^2+^ cytotoxicity to CHO cells was evaluated using cell clonogenic assay after cell treatment with US alone, in the absence of MBs. The impact of different Ca^2+^ concentration (0–20 mM) on cell viability (without US treatment, 0 kPa) as well as after US treatment (400 and 800 kPa) is presented in Figure 3.

Clonogenic assay results have shown that CHO cell incubation with Ca^2+^ without US irradiation (0 kPa) in the range of concentration of 0–20 mM had no effect on cell colony formation efficiency. Similarly, the combined 1–20 mM Ca^2+^ and US treatment of 400 kPa acoustic pressure had no significant effect on cell colony formation efficiency, compared to cell to exposure to US (400 kPa) alone with no administered Ca^2+^ (0 mM Ca^2+^ concentration). The only significant difference (*p* < 0.05) between (US) and (Ca^2+^ + US) groups was observed at 20 mM Ca^2+^ concentration using 800 kPa US acoustic pressure. This indicates the negative effect of Ca^2+^ and US combination for cell biological conditions. 

Since 800 kPa, US had reduced cell colony formation efficiency due to US alone (at 0 mM Ca^2+^ concentration), lower Ca^2+^ concentrations, 0.1 and 0.5 mM, were explored at 800 kPa. However, they had no positive effect for cell colony formation efficiency as compared to (US) group. Although, there is evidence that low Ca^2+^ concentrations facilitate pore closure, therefore increasing cell viability [3,4]. Overall, 400 kPa acoustic pressure had no significant effect on the decrease in cell colony formation efficiency as compared to control (0 mM) in either Ca^2+^ concentration combined with US treatment. Therefore, 400 kPa was selected for further investigation with MBs, as it would better explicit the effect of MB cavitation for Ca^2+^ intracellular delivery.

### 3.2. Ca^2+^ Delivery Using US and MB Combination

**Figure 4:**
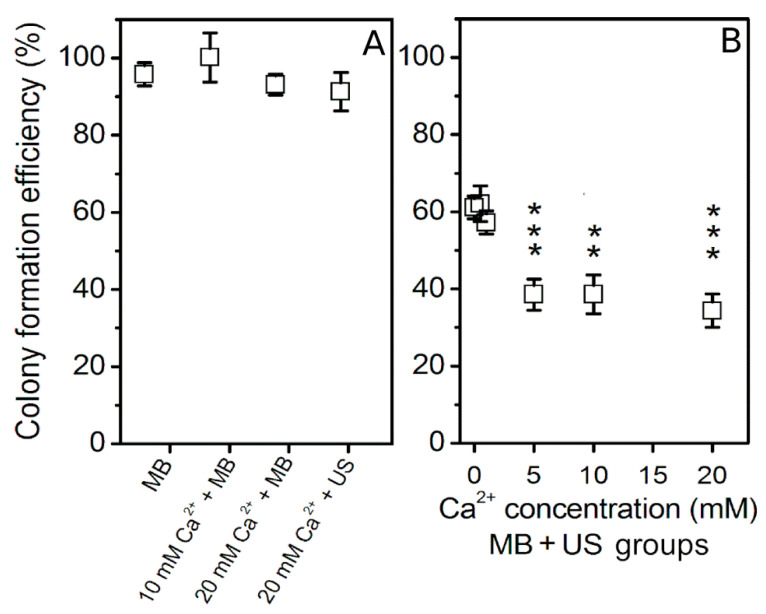
Cell colony formation efficiency, evaluated after cell treatment with US (400 kPa) in the presence of MBs at different Ca^2+^ concentration. (MB), (Ca^2+^ + MB) and (Ca^2+^ + US) experimental groups (**A**); (Ca^2+^ + MB + US) experimental groups (**B**). ** *p* < 0.01, *** *p* < 0.001 indicate statistical significance between cavitation [(Ca^2+^ + MB +US) at 0 mM Ca^2+^ concentration] and therapeutic [(Ca^2+^ + MB +US) at 1–20 mM Ca^2+^ concentration] groups.

The impact of different Ca^2+^ concentration on cell colony formation efficiency was evaluated after MB + US application using 400 kPa acoustic pressure treatment (Figure 4). 

The results of cell clonogenic assay have revealed that 5–20 mM Ca^2+^ concentration in combination with MBs after US treatment have significantly reduced cell colony formation efficiency as compared to the cavitation (MB + US) group, which refers to (Ca^2+^ + MB + US) group at 0 mM Ca^2+^ concentration. In comparison to the cavitation group, lower 0.5 and 1 mM Ca^2+^ concentrations in combination with MBs and US neither reduced cell viability nor had positive effect on cell viability.

**Figure 5:**
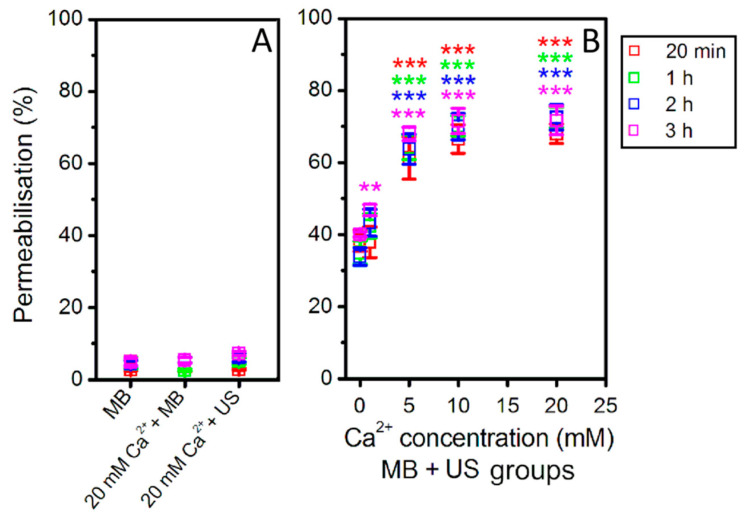
Cell membrane permeabilization (PI assay after 20 min–3 h), evaluated after US (400 kPa) treatment in the presence of MBs at different Ca^2+^ concentration. (MB), (Ca^2+^ + MB) and (Ca^2+^ + US) experimental groups (**A**); (Ca^2+^ + MB + US) experimental groups (**B**). ** *p* < 0.01, *** *p* < 0.001 indicate statistical significance between cavitation [(Ca^2+^ + MB +US) at 0 mM Ca^2+^ concentration] and therapeutic [(Ca^2+^ + MB +US) at 1–20 mM Ca^2+^ concentration] groups.

**Figure 6:**
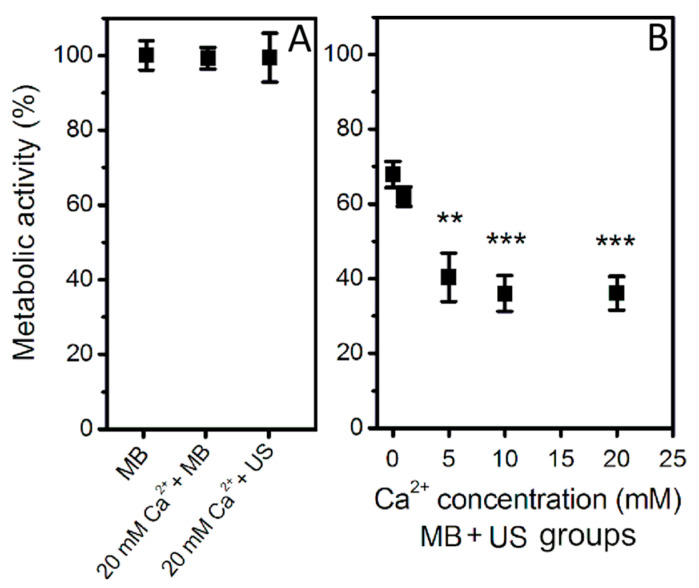
Cell metabolic activity (MTT assay after 48 h), evaluated after US (400 kPa) treatment in the presence of MBs at different Ca^2+^ concentration. (MB), (Ca^2+^ + MB) and (Ca^2+^ + US) experimental groups (**A**); (Ca^2+^ + MB + US) experimental groups (**B**). ** *p* < 0.01, *** *p* < 0.001 indicate statistical significance between cavitation [(Ca^2+^ + MB +US) at 0 mM Ca^2+^ concentration] and therapeutic [(Ca^2+^ + MB +US) at 1–20 mM Ca^2+^ concentration] groups.

In order to gain deeper understanding in cell death dynamics after Ca^2+^ delivery via SP, the impact of different Ca^2+^ concentrations on cell membrane permeabilization and cell metabolic activity were evaluated after (MB + US) application (Figure 5 and Figure 6).

PI assay represents the increase in cell membrane permeability induced by SP (Figure 5). PI was added at a different time after SP: 20 min, 1 h, 2 h, and 3 h. The experimental results show that MBs without US or MB and Ca^2+^ (20 mM) coadministration without US as well as cell exposure to US in the presence of Ca^2+^ (20 mM) have only insignificant effect to cell membrane permeabilization (Figure 5A). The obtained results in (Ca^2+^ + MB + US) groups (Figure 5B) have shown that in the presence of 5–20 mM Ca^2+^ concentration, cell membrane was significantly permeabilized in comparison to cavitation (MB + US) group. US assisted MB cavitation increases membrane permeabilization to ~35–40%, while increasing Ca^2+^ concentration to 5 mM leads to ~60–70% permeabilization, which is similar at higher Ca^2+^ concentrations. The measurements, performed at different PI administration times, indicate (Ca^2+^ + MB + US) induced membrane permeabilization to reach the state of maximal permeabilization within 20 min after treatment, with no significant increase at later time points. The latter finding suggests rapid distribution of highly diffusible calcium ions within the cell interior, eventually leading to directly or indirectly induced cell membrane permeability increase.

In order to find out how these conditions affect cell metabolic activity, MTT assay was performed after 48 h (Figure 6). Similarly, MBs without US or MB and Ca^2+^ (20 mM) coadministration without US as well as cell exposure to US in the presence of Ca^2+^ (20 mM) have only insignificant effect on cell metabolic activity (Figure 6A). However, the combination of US, MB, and Ca^2+^ in the range of 5–20 mM concentration has significantly reduced cell metabolic activity down to ~40% (Figure 6B). The results also indicate that the increase in Ca^2+^ concentration has gradual negative effect for cell metabolic activity.

**Figure 7:**
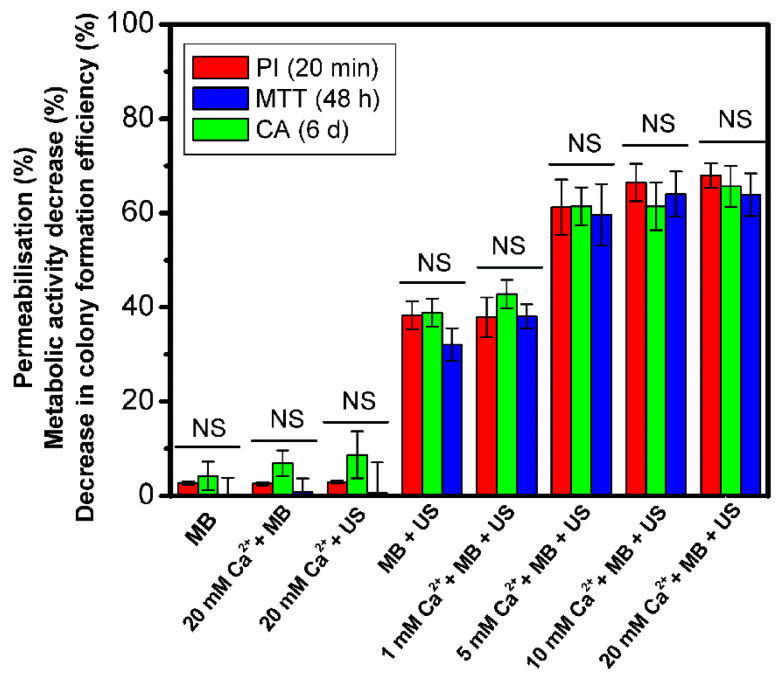
Cell membrane permeabilization, decrease in metabolic activity and decrease in colony formation efficiency, evaluated after SP for different experimental groups and compared within. “NS” indicates no significance between experimental groups (one-way ANOVA).

The overall experimental results of PI, MTT, and colony formation assays are given in Figure 7. The results of membrane permeabilization efficiency, observed after 20 min, are in agreement with the results of decrease in cell metabolic activity (48 h) and cell colony formation efficiency (6 d). Since there was no significant difference (NS) in the results, obtained by different cell viability assays (based on different cell viability characteristics), it implies instant cell death (up to 20 min) to be dominant in Ca^2+^ delivery via SP with no additional cell death occurring within 6 d.

### 3.3. Acoustic Emissions at 400 kPa Acoustic Pressure

**Figure 8:**
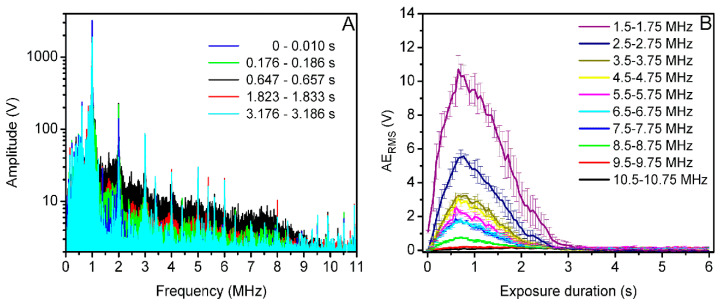
Broadband noise temporal evolution in frequency spectrum (**A**), AE_RMS_ dynamics of the corresponding signal, evaluated in different frequency ranges (**B**), at 400 kPa acoustic pressure US.

Acoustic emission signals, obtained at 400 kPa acoustic pressure, were transformed to frequency spectrum (Figure 8A) for subsequent AE_RMS_ quantification in different frequency ranges from 1.5–1.75 MHz up to 10.5–10.75 MHz (Figure 8B). 

Figure 8A represents the temporal evolution of the amplitude of broadband noise in the frequency domain. As the pronounced frequency components are observed in whole frequency spectrum, reaching their maximal values at 0.647–0.657 s, broadband noise is observed. The latter phenomenon is directly associated to MB inertial cavitation [21,22,23,24,25]. Correspondingly, the curves of AE_RMS_, quantified in different frequency ranges throughout whole frequency spectrum, indicate high above-background acoustic emissions in all of the tested frequency ranges up to 8.5–8.75 MHz (Figure 8B). AE_RMS_ values, quantified in 9.5–9.75 and 10.5–10.75 MHz frequency bands, had minor increases above background level, which is in line with the frequency spectrum. The highest difference in acoustic emissions between +MB and −MB groups was determined in the 1.5–1.75 MHz frequency range. In addition to this, in all the frequency ranges tested, AE_RMS_ decrease to ~0 V background level occurred within 3 s (Figure 8B), indicating complete MB sonodestruction. Thus, the adjusted exposure duration of 6 s is sufficient for maximal sonoporation efficiency.

### 3.4. Sonoporation Results in Acoustic Pressure Scale

**Figure 9:**
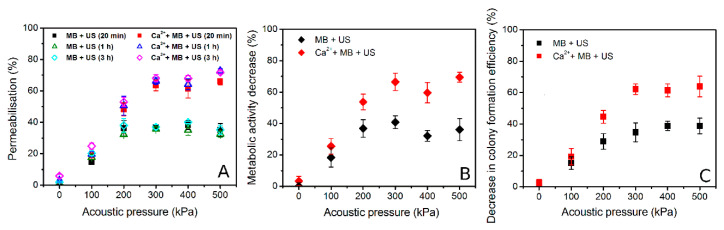
Cell membrane permeabilization (**A**), metabolic activity decrease (**B**) and decrease in cell colony formation efficiency (**C**), evaluated in cavitation (MB + US) and therapeutic (Ca^2+^ + MB + US) groups at different acoustic pressure.

In order to investigate the role of cavitation in Ca^2+^ intracellular delivery via SP, acoustic pressure was varied in the range of 0–500 kPa at constant 5 mM Ca^2+^ concentration. The latter concentration was selected according to the data of previous experiments, as the increase in Ca^2+^ concentration from 5 to 10 or 20 mM had practically no influence on inducing additional cell death in (Ca^2+^ + MB + US) group (Figure 4, Figure 5 and Figure 6). The results of cell permeabilization, metabolic activity decrease, and colony formation activity decrease at constant 5 mM Ca^2+^ concentration and varied acoustic pressure are presented in Figure 9.

Similar dynamics have been observed in the results of cell death, evaluated by different cell viability assays. PI assay results, obtained by PI test at different time after SP (Figure 9A), indicate increasing permeability for both cavitation (MB + US) and therapeutic (Ca^2+^ + MB + US) groups up to 300 kPa with the subsequent plateau of ~35% and ~65%, respectively, achieved at higher acoustic pressures. At constant acoustic pressure, the results of membrane permeabilization, obtained at different times, indicate similar permeabilization efficiency, which remains unchanged from 20 min up to 3 h. The results of decrease in cell metabolic activity (Figure 9B) indicate a similar tendency with increasing acoustic pressure, followed by subsequent saturation at 300–500 kPa for both cavitation (~35%) and therapeutic (~60%) groups. The results of cell clonogenic assay (Figure 9C) are in accordance with the results of previous tests, indicating a decrease in cell colony formation efficiency for both cavitation (MB + US) and therapeutic (Ca^2+^ + MB + US) groups up to 300 kPa, with further saturation in the range of 300–500 kPa at (~35–40%) and (~65%) for the respective groups. As PI, MTT, and cell clonogenic assay results have similar tendencies and values in the acoustic pressure range, we again observe that cell death achieved after 20 min sustains the same after the 48 h as well as 6 d period.

**Figure 10:**
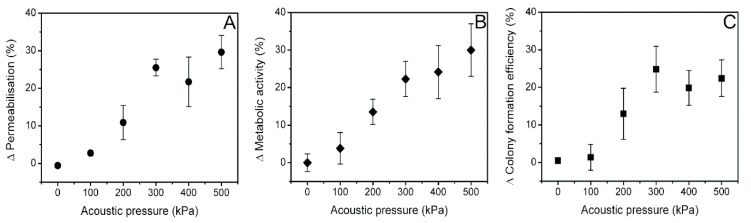
The change (∆) in cell permeabilization (**A**), cell metabolic activity (**B**), and cell colony formation efficiency (**C**), evaluated as permeabilization, decrease in metabolic activity, and decrease in colony formation efficiency between groups: (Ca^2+^ + MB + US)−(MB + US)−(Ca^2+^ + MB).

The results of PI, MTT, and cell clonogenic assays were used to calculate the changes in cell membrane permeability, obtained after 20 min (Δ Permeabilisation) (Figure 10A), metabolic activity (Δ Metabolic activity) (Figure 10B), and colony formation efficiency (Δ Colony formation efficiency) (Figure 10C). Either change was evaluated as the difference in cell membrane permeabilization, decrease in metabolic activity, or decrease in colony formation efficiency of three groups: (Ca^2+^ + MB + US)−(MB + US)−(Ca^2+^ + MB).

Δ Permeabilization, evaluated at standard time after SP, 20 min (Figure 10A), gradually increases following the increment in acoustic pressure until it reaches saturation level of ~25–30% at 300 kPa acoustic pressure. Δ Metabolic activity (Figure 10B) and Δ cell colony formation efficiency (Figure 10C) have similar absolute values and dynamics in acoustic pressure scale as Δ permeabilization.

### 3.5. Acoustic Emission Quantification in Acoustic Pressure Scale 

**Figure 11:**
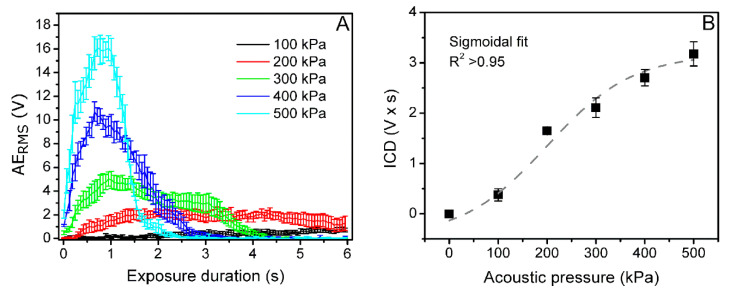
AE_RMS_ curves in exposure duration scale at different acoustic pressure (**A**). ICD in acoustic pressure scale (**B**).

Since the increase in US acoustic pressure is associated to intensification in cavitation activity, MB acoustic emission signals, detected at different acoustic pressures (0–500 kPa), were used to evaluate AE_RMS_ in 1.5–1.75 MHz frequency range. The obtained AE_RMS_ curves in exposure duration scale are given in Figure 11A.

AE_RMS_, plotted in exposure duration scale, represents both the intensity and duration of MB cavitation activity. Thus, the integral combining both parameters is considered to be a direct estimate of MB cavitation. In order to estimate ICD, the integral of AE_RMS_ was calculated during the entire US exposure duration for each acoustic pressure value using Equation (3). The form of ICD curve, plotted in the acoustic pressure range, is sigmoidal (Figure 11B).

### 3.6. ICD and Biological Effect Correlation

**Figure 12:**
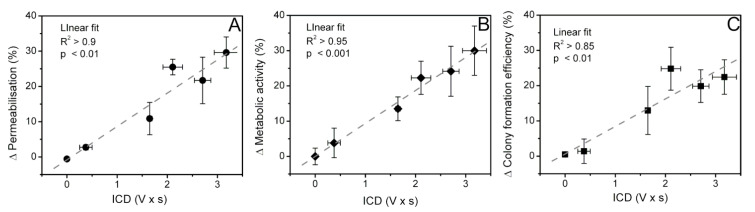
The correlation of ICD and the change (Δ) in cell permeabilization (**A**), cell metabolic activity (**B**), and cell colony formation efficiency (**C**).

The results of Δ permeabilization, Δ cell metabolic activity, and Δ cell colony formation efficiency were plotted in the scale of ICD in order to establish the quantified relation to MB inertial cavitation (Figure 12).

The correlation results of ICD and the change in permeabilization (Figure 12A), metabolic activity (Figure 12B), and cell colony formation efficiency (Figure 12C) indicate strong (R^2^ > 0.85) significant (*p* < 0.05) correlation. This shows that the obtained results, represented by three different cell viability assays, based on different cell viability characteristics: membrane permeabilisation, metabolic activity, and cell colony formation efficiency, are linearly dependent on the “amount” of broadband noise, produced by cavitating MBs, and are directly associated to US induced MB IC.

## 4. Discussion

In the current study, we have employed three different assays to evaluate cell viability dynamics after SP-mediated calcium delivery into CHO cells. They all indicate that Ca^2+^ increase in combination with US assisted MB cavitation negatively affects all three different vital parameters associated to cell viability: cell membrane integrity, cell metabolic activity, and cell colony formation efficiency, in a similar fashion. PI assay has indicated decrease in membrane integrity after 20 min, which sustained similar for up to 3 h; therefore, estimated pore lifetime was at least—3 h. The results of MTT and cell clonogenic assays have shown similar tendency and level as PI values. Therefore, we have obtained the same cell death level after 20 min (PI assay) compared to 24 h (MTT assay) as well as to 6 d (cell clonogenic assay). This indicates rapid cell death due to Ca^2+^ delivery via SP. In contrast to other studies [3,4] we have not observed a positive effect of low (up to 3 mM) Ca^2+^ concentration for cell viability neither for pore resealing nor cell viability. The detailed reasoning behind this is to be determined in future research. 

The viability dynamics of CHO cells after Ca^2+^ delivery via SP are very similar to those obtained in our previous works on BLM delivery [26,27]. Similarly, we have attained efficient Ca^2+^ delivery via SP only after combined US–MB employment, as we have observed no significant changes in cell viability after 6 d due to the application of US alone, except at 800 kPa using 20 mM Ca^2+^ concentration. In addition to this, the efficiency of BLM delivery was achieved ~30%, evaluated by cell clonogenic assay, and it is line with the current results using Ca^2+^. What is more, the mechanism for both BLM and Ca^2+^ intracellular delivery was associated to MB IC. These findings indicate a similar anticancer agent delivery mechanism leading to analogous outcome, and indirectly imply successful future feasibility of Ca^2+^ delivery via SP to initiate anticancer activity in tumor cells.

The activity of anticancer chemotherapy and gene therapy is rather slow in cell death, since the effect of anticancer drug use was achieved after 24–48 h [5,6,7,8,10] and plasmid DNA internalization to nucleus and expression takes long durations so that the maximum level of gene expression efficiency was generally obtained after 12–72 h [16,17,18,19]. In contrast to the conventional therapy techniques, rapid Ca^2+^ distribution within the cell interior via SP alters the vital state of the cell by intrinsic processes and induces a rapid cell killing effect. Therefore, Ca^2+^ application for anticancer treatment would offer new opportunities for initiating the rapid cell killing effect, occurring within 20 min. This also reduces high systemic cytotoxicity imposed by anticancer drugs. 

SC is characterized by linear volumetric oscillations of MBs at lower driving pressures, while IC dominates at higher pressures and signifies in non-linear MB vibrations culminating in implosion. The spectral content of emitted US waves during SC consists of harmonic, subharmonic, and ultraharmonic constituents of US central frequency. Intense MB collapse generates white noise with components plainly observed in all the frequencies of the spectrum of analyzed output US signals. Therefore, broadband noise is considered to be an unambiguous confirmation of MB collapse and, respectively, IC [25,28,29]. IC has been substantiated to be involved in SP according to high correlation of IC based metrics, MB sonodestruction rate [26] and inertial cavitation dose [21], with the efficacy of therapeutic agent sonotransfer [23,24,26,30] and cell viability [21,23,26]. Therefore, passively registered acoustic emissions from MBs, induced by US, were analyzed in order to acquire valuable information about cavitation pattern. After initial analysis, the amount of broadband noise was quantified as differential RMS in different frequency ranges with the aim to obtain the estimate “AE_RMS_”. IC presence (at 400 kPa acoustic pressure) was indicated by temporal curves of AE_RMS_ estimate, ranging above 0 V baseline in all frequency ranges 1.5–1.75 to 8.5–8.75 MHz. The 1.5–1.75 MHz frequency range was empirically associated to the highest broadband noise emissions according to the highest difference between +MB and −MB groups. Therefore, the integral metric, ICD, was evaluated in this range for the subsequent correlation analysis in the acoustic pressure scale.

Since the beginning of investigation in the mechanism behind SP, an ongoing discourse has been initiated by the researchers about SC vs. IC [20]. SC employment for SP is considered to be very convenient, as it allows the attainment of maximal site-specificity of the method without imposing damage to nearby healthy cells. Low bioeffect efficiency, obtained at low acoustic pressure, 100 kPa, was the preliminary indication of the necessity of IC in order to induce effective SP. Specific evidence of IC presence in Ca^2+^ delivery via SP are the AE_RMS_ curves, which quantitatively indicate the presence of broadband noise in the 1.5–1.75 to 8.5–8.75 MHz frequency ranges. The registered broadband noise emissions of cavitating MBs are considered to be the distinctive feature of IC and allow the detection and quantification of IC activities [21,22,23,24,25]. 

The cumulated amount of broadband noise, ICD, highly correlates (R^2^ > 0.85) with the results, provided by different cell viability assays: (i) membrane permeabilization (PI assay), metabolic activity (MTT assay), and colony formation efficiency (cell clonogenic assay). These relations indicate the cellular bioeffects of different origin, produced by Ca^2+^ delivery via SP, to be directly associated to IC activity. It also implies that Ca^2+^ delivery via MB IC alters all three different cell viability criteria, independently estimated by three different cell viability tests. 

The role of SC in SP becomes dominant when MBs are positioned near or bound to the cell surface; thereby, the distance between the cell and nearby MBs is reduced [31,32]. Since the impact of microstreaming is significantly downscaled with the increment in cell–MB separation, SC intensity is reduced and, therefore, IC prevails [22,23,24,26,27,30]. The assumption is reinforced by effective SP, obtained at SC regime, conducted on monolayers, where cells are tightly embedded and come into close contact with adjacent MBs [33,34].

In general, sudden cell death by Ca^2+^ delivery via SP is associated to MB IC, as it was empirically revealed by high ICD correlation with change in either permeabilization, metabolic activity, or colony formation efficiency. Therefore, findings, obtained in our study, indicate that in order to initiate rapid cell killing via Ca^2+^ delivery via SP, the infliction of MB IC activity is preferred.

## 5. Conclusions

1. The increase in Ca^2+^ concentration during sonoporation negatively affects the fundamental characteristics of cell viability: (i) cell membrane integrity, (ii) cell metabolic activity, and (iii) cell colony formation efficiency, which were independently evaluated using different cell viability assays.
2. Ca^2+^ delivery via sonoporation induces instantly occurring cell death—within 20 min after treatment, with no additional cell death observed within the 2-d to 6-d period.
3. High correlation (R^2^ > 0.85, *p* < 0.01) of inertial cavitation dose with the results of different-criterion-based cell viability assays implies inertial cavitation activities to be directly involved in calcium delivery via sonoporation.

## Data Availability

The data supporting the findings of this study are available within the article.
